# Immunization coverage in Ethiopia among 12–23 month old children: systematic review and meta-analysis

**DOI:** 10.1186/s12889-020-09118-1

**Published:** 2020-07-20

**Authors:** Tahir Yousuf Nour, Alinoor Mohamed Farah, Omer Moeline Ali, Kalkidan Hassen Abate

**Affiliations:** 1 Department of Public Health, College of Medicine and Health Science, Jigjiga University, Jigjiga, Ethiopia; 2 Department of Population and Family Health, Institute of Health Sciences, Jimma University, Jimma, Ethiopia

**Keywords:** Immunization coverage, Systematic review, Pastoral, Semi-pastoral, Meta-analysis, Ethiopia

## Abstract

**Background:**

Immunization is a cost-effective public health strategy. Immunization averts nearly three million deaths annually but immunization coverage is low in some countries and some regions within countries. The aim of this systematic review and meta-analysis is to assess pooled immunization coverage in Ethiopia.

**Method:**

A systematic search was done from PubMed, Google Scholar, EMBASE, HINARI, and SCOPUS, WHO’s Institutional Repository for Information Sharing (IRIS), African Journals Online databases, grey literature and reviewing reference lists of already identified articles. A checklist from the Joanna Briggs Institute was used for appraisal. The I^2^ was used to assess heterogeneity among studies. Funnel plot were used to assess publication bias. A random effect model was used to estimate the pooled prevalence of immunization among 12–23 month old children using STATA 13 software.

**Result:**

Twenty eight articles were included in the meta-analysis with a total sample size of 20,048 children (12–23 months old). The pooled prevalence of immunization among 12–23 month old children in Ethiopia was found to be 47% (95%, CI: 46.0, 47.0). A subgroup analysis by region indicated the lowest proportion of immunized children in the Afar region, 21% (95%, CI: 18.0, 24.0) and the highest in the Amhara region, 89% (95%, CI: 85.0, 92.0).

**Conclusion:**

Nearly 50% of 12–23 month old children in Ethiopia were fully vaccinated according to this systematic review and meta-analysis this indicates that the coverage, is still low with a clear disparity among regions. Our finding suggests the need for mobile and outreach immunization services for hard to reach areas, especially pastoral and semi-pastoral regions. In addition, more research may be needed to get more representative data for all regions.

**PROSPERO registration number:**

CRD42020166787.

## Background

Immunization is one of the cost-effective public health interventions, and can be used to limit life threatening childhood illnesses like diphtheria, pertussis, tetanus, hepatitis virus, measles, mumps, pneumonia, polio virus and rotavirus [1, 2].

Worldwide Expanded Program of Immunization (EPI) was launched in 1974 to all WHO members with the aim of strengthening routine immunization coverage and decreasing Vaccine Preventable Diseases (VPDs). In Ethiopia routine immunization was started in 1980 with a purpose of reducing maternal and child morbidity and mortality [3–5].

Globally, immunization averts 2–3 million deaths yearly, but VPDs still accounts worldwide for over 2 million under five deaths annually, the majority of them being from countries in sub-Saharan Africa [6].

A 2017 WHO report revealed that, 116.2 million infants (85%) received a third dose of DPT globally, only 123 (63%) of 194 countries achieved three doses of DPT coverage ≥90% [7–9].

A 2018 WHO report found 13.5 million infants had never received any dose of vaccine, and 19.4 million never received the third dose of DPT. Among all children who were under and unimmunized, 11.7 million lived in just ten countries one of which is Ethiopia [10].

At present, vaccination is being given on static, outreach and mobile basis [11]. Ethiopia follows WHO recommended immunization schedule. Infants should receive vaccination in 1 year after birth with: one dose bacillus Calmette–Guerin (BCG) and oral polio vaccine is given at birth or as soon as possible; three doses of OPV, three doses of Penta-valent, three doses pneumococcal vaccines, two doses of rotavirus are given at interval of 4 weeks duration at the 6th, 10th and 14th weeks, 9 month first dose of measles [11]. The second dose of measles vaccine was launched and integrated to the routine immunization in the year of 2019 and which was provided to 15- month old children [12].

Of these vaccines, the third dose of DPT (DPT 3) is often used as a measure of immunization system performance. Instead of DPT, some countries use a Penta-valent vaccine, which includes (Diphtheria, Pertussis, Tetanus, Heamophilus influence type B and Hepatitis B) [11].

The government has shown strong commitment to improving EPI access and utilization by training nearly four thousand health extension workers and setting up 15,000 health posts which have integrated community case management in 2010 [11, 13]. It is also endorsing sustainable development goal target 3.2 which is aimed at reducing preventable deaths of newborns and under-five children by 2030 [14].

Despite increased trend of immunization coverage in the last decades [15, 16], the most common VPDs that substantially result in under-five morbidity and mortality are pneumonia, diarrhea and measles [17, 18]. Pneumonia is a leading single killer of under-five children [19]. It is estimated that 3,370,000 children encounter pneumonia annually in Ethiopia which contributes to 18% of all causes of deaths and claiming over 40,000 under-five children every year [17]. Diarrhea is the second leading cause of under-five mortality with an estimated 1.7 million cases of diarrhea annually [19]. Morbidity reports and community-based studies indicated diarrheal disease is still public health problem [20–22]. Likewise, measles is one of the public health problems with an incidence of 50 cases/1,000,000 population per year which is above the national target for measles elimination by 2020 (1 < cases /1,000,000 population per year). The estimated case fatality is between 3 and 6% but this rate is underestimated because of incomplete reporting of measles [12].

In Ethiopia, all health delivery services are decentralized in to 11 self-administering regions and two city administrations. They provide healthcare services from Zonal to Keble level (the smallest administrative unit) they get vaccines as per their request from EPSA (Ethiopia Pharmaceutical Supply Agency) which is an autonomous agency of FMoH with responsibility of policy, direction setting and vaccine distribution with support of UNICEF for forecasting, procurement, and overall technical support with adequate vaccine supply [23].

The cold chain system is highly sensitive to any kind of mishandling and power interruption. FMoH and partners implemented and endorsed cold chain rehabilitation plan, regular cascade training of cold chain technicians, national cold chain inventory and installing solar fridge for hard to reach area were done but still there is no published articles for cold chain at all level [23, 24].

Numerous studies revealed that rural residence, government employer, female household head, parents having formal education of primary and above, antenatal care follow up, giving birth at health facility, good knowledge about immunization benefit and schedule, short distance to health facility, having four or more family size can increase vaccine coverage contrary to fear of side effects, low wealth status, being too busy, lack of awareness about vaccination, poor perception toward vaccination, were predictors of child immunization [7, 25–28].

Understanding the extent of vaccine coverage and its associated factors are important for designing strategies that can reduce the burden of vaccine preventable diseases. There is no systematic review and meta-analysis conducted in Ethiopia on immunization coverage among children 12–23 month old. Therefore, aims of this was to pool and synthesize recent evidence on the prevalence of vaccine coverage among 12–23 months old children using small fragmented regional studies to pool and form national result to consolidate available data and determine current prevalence of vaccine coverage in Ethiopia.

## Methods

### Protocol and registration

This systematic review and meta-analysis was registered with international prospective register and systematic reviews PROSPERO (2020 CRD42020166787) (https://www.crd.york.ac.uk/prospero/display_record.php?ID=CRD42020166787.

### Search strategy and appraisal of studies

All published studies conducted in Ethiopia reporting the fully vaccinated or immunization coverage from September 2009 to 2019 were included. The search was limited to English language, human and cross-sectional studies. This study was conducted according to the Preferred Reporting Items of Systematic Reviews and Meta-Analysis Protocols (PRISMA) checklist guideline [29]. The electronic databases searched were PubMed, Google Scholar, EMBASE, HINARI, SCOPUS, WHO’s Institutional Repository for Information Sharing (IRIS), and African Journals Online databases. In addition to that, articles were also searched through a review of the grey literature available on local universities repository and by reviewing the reference lists of already identified articles. The key terms used for the database searches were connected by Boolean operators in our search below Medical Subject Headings (MeSH terms) and combined as follows: “Epidemiologic” OR “Child” OR “Children” AND “Coverage, Vaccination” OR “Vaccination coverages” OR “Immunization Coverage” OR “Coverage, Immunization” OR “coverage’s, Immunization” OR “Immunization coverage” AND “Ethiopia” Filters: Free full text; Publication date from 2009/01/01 to 2019/12/31; Humans Sort by: [pubsolr12]. The searched articles were publication from 01/01/2009 to 01/11/2019.

### Inclusion and exclusion criteria

#### Inclusion

**Study area:** only those studies conducted in Ethiopia were considered as included studies.

**Participants/population**: Children 12–23 month old were included.

**Intervention:** Immunization for eligible children (< 2 year).

**Comparison**: those immunized and those who were unimmunized.

**Study design:** observational studies (cross-sectional were included).

All English-language, full-text articles conducted in Ethiopia, published from January 2009 to September 2019, in peer-reviewed journals or grey literature was eligible for inclusion criteria.

#### Exclusion

Studies that did not report specific outcomes for immunization coverage quantitatively, age of children greater than 24 month old and study design other than cross-sectional study were excluded.

### Outcome measurement

Primary outcome of this study was prevalence of immunization coverage among 12–23 month old children in Ethiopia. It was measured as the number of children who were fully immunized divided by the total number of children in studies multiplied by 100.

Case definition of fully immunized children is when they have received one dose of Bacillus Calmette Guerin (BCG), three doses of DPT, three doses of polio vaccines and one dose of measles vaccination by the age of 9–12 months [11]. All studies that reported the vaccination coverage in children aged 12–23 month were eligible for inclusion.

### Data abstraction procedure

Two authors (TY and AM) independently extracted data using Joanna Briggs Institute data extraction tool for applied cross-sectional study design. Any disagreement was resolved through discussion and consensus reached through third person (KHA). Outcome data extraction format contains author, publication year, region, sample size, study design, response rate, and prevalence with 95% confidence interval. Retrieved articles were evaluated based on their title, objective and methodology. At this stage, irrelevant articles were excluded and the full texts of the remainder articles were reviewed for inclusion criteria. Articles that fulfilled the prior criteria were used as a source of data for the final analysis.

### The database search results were exported

Duplicate articles were removed using EndNote software (version X7; Thomson Reuters, New York, NY). Two independent reviewers critically appraised each individual article using the quality assessment checklist for prevalence studies before analysis which was included representation, sampling, random selection, non-response rate, data collection, case definition, reliability and validity of study tool, method of data collection, prevalence,numerator and denominator was assessed for quality summary [30]. It was categorized as having low risk of bias “yes” high risk of bias “no” if the score is 8–10 we consider it as having “low risk of bias” 6–7 score “moderate risk of bias” 0–5 score “high risk of bias”(Table 1) (below).

**Table 1:** Table 1 Risk of bias assessment of eligible articles using the Hoy 2012 tool

study	Representation	sampling	Random selection	Non response rate	Data collection	Case definition	Reliability and Validity	Method of Data collection	prevalence	Numerator and Denominator	summary Assessment
Animaw et al. [31]	High risk	Low risk	Low risk	Low risk	Low risk	Low risk	Low risk	Low risk	Low risk	Low risk	10
Etana and Deressa [32]	High risk	Low risk	Low risk	Low risk	Low risk	Low risk	Low risk	Low risk	Low risk	Low risk	9
Gualu and Dilie [33]	High risk	Low risk	Low risk	Low risk	Low risk	Low risk	Low risk	Low risk	Low risk	Low risk	9
Hailu et al. [8]	Low risk	Low risk	Low risk	Low risk	Low risk	Low risk	Low risk	Low risk	Low risk	Low risk	10
Girmay and Dadi [25]	Low risk	Low risk	Low risk	Low risk	Low risk	Low risk	Low risk	Low risk	Low risk	Low risk	10
Kassahun et al. [34]	High risk	Low risk	Low risk	Low risk	Low risk	Low risk	Low risk	Low risk	Low risk	Low risk	9
Lake et al. [35]	High risk	High risk	Low risk	Low risk	High risk	Low risk	High risk	Low risk	Low risk	Low risk	6
Legesse and Dechasa [36]	Low risk	Low risk	Low risk	Low risk	Low risk	Low risk	High risk	Low risk	Low risk	Low risk	9
Mekonnen et al. [37]	Low risk	Low risk	Low risk	Low risk	Low risk	Low risk	Low risk	Low risk	Low risk	Low risk	10
Meleko et al. [38]	High risk	Low risk	Low risk	Low risk	Low risk	Low risk	Low risk	Low risk	Low risk	Low risk	9
Mohammed and Atomsa [39]	High risk	Low risk	Low risk	Low risk	Low risk	Low risk	High risk	Low risk	Low risk	Low risk	8
Mohamud et al. [40]	Low risk	Low risk	Low risk	Low risk	Low risk	Low risk	High risk	Low risk	Low risk	Low risk	9
Tefera et al. [27]	High risk	Low risk	High risk	Low risk	Low risk	Low risk	High risk	Low risk	Low risk	Low risk	7
Fite and Haili [41]	High risk	High risk	Low risk	Low risk	High risk	Low risk	High risk	Low risk	High risk	High risk	4
Tesfaye et al. [42]	Low risk	Low risk	Low risk	Low risk	Low risk	Low risk	High risk	Low risk	Low risk	Low risk	9
Wado et al. [43]	High risk	Low risk	Low risk	Low risk	Low risk	Low risk	High risk	Low risk	Low risk	Low risk	7
Yismaw et al. [44]	Low risk	Low risk	Low risk	Low risk	Low risk	Low risk	High risk	Low risk	Low risk	Low risk	9
Beyene et al. [45]	High risk	Low risk	Low risk	Low risk	High risk	Low risk	High risk	Low risk	Low risk	Low risk	7
Mebrahtom and Birhane [46]	High risk	Low risk	Low risk	Low risk	Low risk	Low risk	Low risk	Low risk	Low risk	Low risk	9
Debie and Taye [47]	High risk	Low risk	Low risk	Low risk	Low risk	Low risk	High risk	Low risk	Low risk	Low risk	7
Facha W [48]	High risk	Low risk	Low risk	Low risk	Low risk	Low risk	High risk	Low risk	Low risk	Low risk	8
Ebrahim and Beyene [49]	High risk	Low risk	Low risk	Low risk	Low risk	Low risk	Low risk	Low risk	Low risk	Low risk	9
Tessema et al. [50]	High risk	Low risk	Low risk	Low risk	Low risk	Low risk	Low risk	Low risk	Low risk	Low risk	9
Kidane et al. [51]	High risk	Low risk	Low risk	Low risk	Low risk	Low risk	Low risk	Low risk	Low risk	Low risk	9
Porth et al. [52]	High risk	Low risk	High risk	Low risk	High risk	Low risk	High risk	Low risk	Low risk	Low risk	6
EDHS [53]	Low risk	Low risk	Low risk	Low risk	Low risk	Low risk	Low risk	Low risk	Low risk	Low risk	10
EDHS [16]	Low risk	Low risk	Low risk	Low risk	Low risk	Low risk	Low risk	Low risk	Low risk	Low risk	10
EDHS [15]	Low risk	Low risk	Low risk	Low risk	Low risk	Low risk	Low risk	Low risk	Low risk	Low risk	10

Any disagreement between reviewers was resolved through discussion and reaching consensus including a third reviewer. The average of two independent reviewer’s quality scores were used to determine whether the articles should be included. Articles with methodological errors or incomplete reporting of results with no full text were excluded from the final analysis.

### Statistical analysis

The aim of this review was to assess pooled prevalence of immunization coverage among 12–23 month old children in Ethiopia.

Information on the studies characteristics such as publication year, study region, study design, sample size, non-respondent and immunization coverage were extracted from each study using a Microsoft Excel spreadsheet template. Then, data were exported to Stata (version 13; Stata Corp, College Station, TX) for further analysis.

A random effect model was used to estimate pooled prevalence of fully vaccinated children with 95% confidence interval (CI) in Ethiopia. Funnel plot asymmetry was used to check for publication bias (Fig. 4). Heterogeneity between studies was assessed using I-squared (I^2^) statistics and the cutoffs of 25, 50, 75% was used to declare heterogeneity as low, moderate and high respectively [54]. Subgroup analysis was also conducted to detect prevalence of immunization coverage and heterogeneity among regional states of the country.

### Operational definitions

**Pastoralist and semi-pastoralist**: means communities, located in some part of Benishangul Gumuz, Gambella, Oromia, Somali, and Southern Nations, Nationalities and Peoples’ region [51].

**Fully vaccinated**: when children receive one dose of Bacillus Calmette Guerin (BCG), three doses of DPT, three doses of polio vaccines, and one dose of measles vaccination by the age of 9–12 months [11].

## Result

**Fig. 1:**
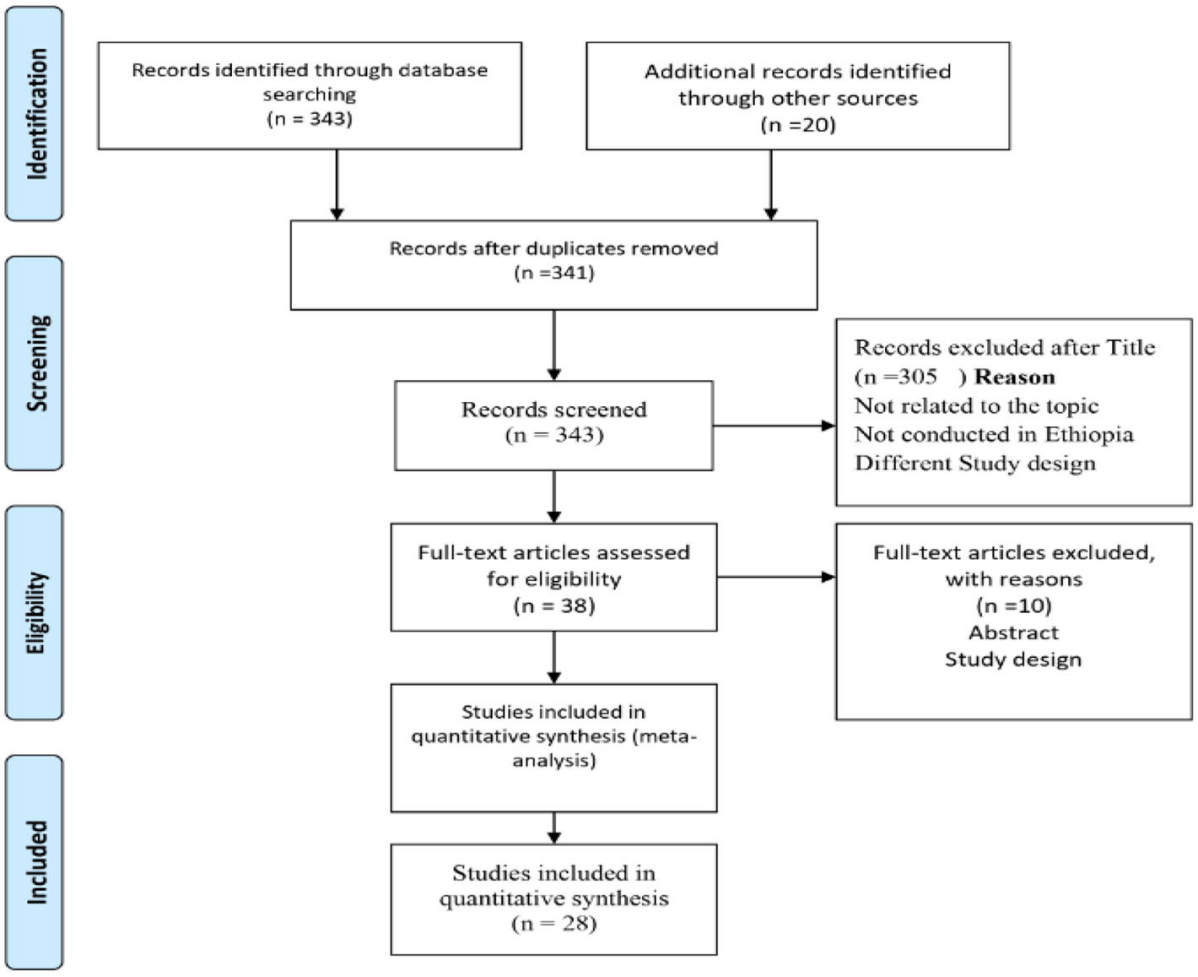
PRISMA Flow showing searching strategies of studies for systematic review and meta-analysis of immunization coverage among 12–23 month old children in Ethiopia

In the first step of our search, 363 articles were retrieved from immunization coverage among children aged 12–23 months using different electronic databases were searched: like PubMed, Google Scholar, EMBASE, HINARI, Web of Science, SCOPUS, WHO’s Institutional Repository for Information Sharing (IRIS), and African Journals Online databases. 22 articles were excluded due to duplication, from remaining 341 articles 308 articles were excluded as they were not related to the topic, not done in Ethiopia and study design. Only 35 full text articles were accessed and assessed for their eligibility based on inclusion criteria. Finally 28 articles were eligible and included in this systematic review and meta-analysis (Fig. 1) (below).

### Characteristics of included studies

All retained twenty eight studies in this systematic review and meta-analysis conducted in Ethiopia were cross-sectional study; twenty five of them were conducted from eight regional states and three national surveys, a total sample size of 20,048 children aged 12–23 months were included. The minimum sample size was 173 [41] and maximum sample size was 2004 [16]. Nin (32.1%) of studies were from Amhara region [31, 33–35, 37, 42, 44, 47, 49], six (21.4%) from South Nation Nationality and People’s region [8, 27, 38, 41, 48, 52], four (14.3%) from Oromia region [32, 36, 39, 43], were three (10.7%) survey nationwide [15, 16, 55], two (7.1%) from pastoral and semi-pastoral area of the country [50, 51], lastly one (3.6%) from Somali state [40] and one (3.6%) from Tigrai regional states [25]. All studies were published in peer review journals. Fully vaccinated criteria were based on maternal recall; immunization card and BCG scar.

**Fig. 2:**
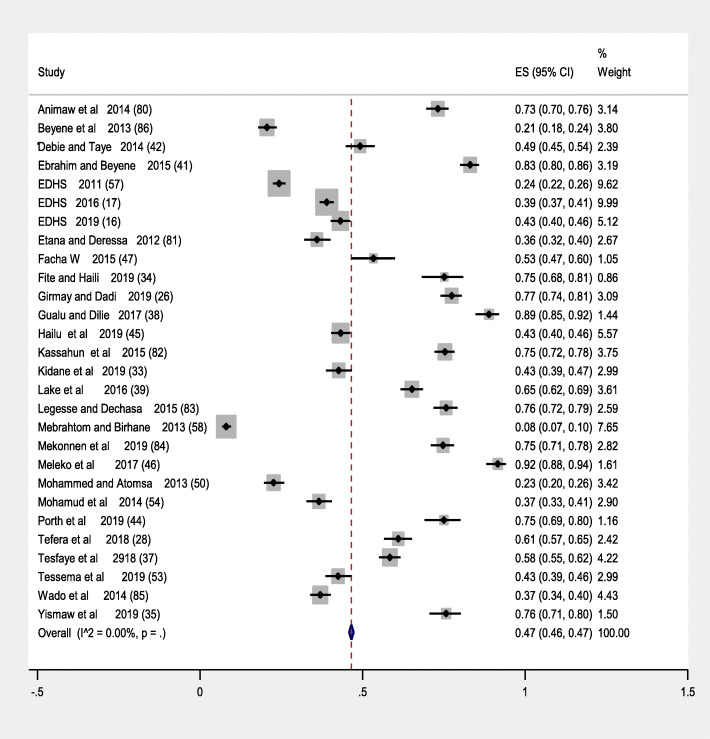
Forest plot pooled prevalence of immunization coverage in Ethiopia from 2009 to 2019

An estimated over all pooled prevalence of immunization coverage among 12–23 month old children in Ethiopia was 47% (95% CI: 46.0, 047.0). The lowest proportion included studies was 21% (95%,CI:18.0,24.0) [45] and the highest 89% (95%,CI:85.0,92.0) [33]. The I-square test showed that there was no heterogeneity among included studies (I^2^ = 0.00, *p*-value < 0.005). Studies with largest weight were EDHS 2016 [16], EDHS 2011 [53] and Mebrahtom and Birhane [46] 9.99,9.62,7.65 respectively. While those with smallest weight were from Facha wolde [48], Porth et al. [52] and Gualu and Dillie [33] respectively 1.05, 1.16 and 1.44 (Table 2) (below). Using random effect model analysis the pooled prevalence of immunization coverage among 12–23 moths in Ethiopia showed (I^2^ = 0.000, p-value< 0.005) (Fig. 2) (below).

**Table 2:** summary characteristics of included studies in meta-analysis of immunization coverage in Ethiopia, 2009 to 2019

Author	year	Region	study base	Event	N	Response rate	Quality score	prevalence with it is 95% CI
Animaw et al. [31]	2014	Amhara	community based	461	630	100%	9	73% (70,76)
Etana and Deressa [32]	2012	Oromia	community based	193	536	100%	9	36% (32,40)
Gualu and Dilie [33]	2017	Amhara	community based	256	288	96.80%	9	89% (85,92)
Hailu et al. [8]	2019	SNNP	community based	483	1116	82.70%	10	43% (40,46)
Girmay and Dadi [25]	2019	Tegrai	community based	480	620	99.50%	10	77% (74,81)
Kassahun et al. [34]	2915	Amhara	community based	566	751	99.20%	9	75% (72,78)
Lake et al. [35]	2016	Amhara	community based	472	724	100%	6	65% (62,69)
Legesse and Dechasa [36]	2015	Oromia	community based	393	519	98.50%	9	76% (72,79)
Mekonnen et al. [37]	2019	Amhara	community based	423	566	98.80%	10	75% (71,78)
Meleko et al. [38]	2017	SNNP	community based	295	322	100%	9	92% (88,94)
Mohammed and Atomsa [39]	2013	Oromia	community based	155	685	98.70%	8	23% (20,26)
Mohamud et al. [40]	2014	Somali	community based	213	582	100%	9	37% (33,41)
Tefera et al. [27]	2018	SNNP	community based	295	484	89.60%	7	61%(57,65)
Fite and Haili [41]	2019	SNNP	community based	130	173	100%	4	75%(68,81)
Tesfaye et al. [42]	2018	Amhara	community based	494	846	98.11%	9	58% (55,62)
Wado et al. [43]	2014	Oromia	community based	329	889	100%	7	37% (34,40)
Yismaw et al. [44]	2019	Amhara	community based	228	301	100%	9	76% (71,80)
Beyene et al. [45]	2013	Afar	community based	157	762	97.10%	7	21% (1824)
Mebrahtom and Birhane [46]	2013	Afar	community based	124	1534	98.30%	9	8% (7.0,10)
Debie and Taye [47]	2014	Amhara	community based	236	479	100%	7	49% (45,54)
Facha W [48]	2015	SNNP	community based	112	210	100%	8	53% (47,60)
Ebrahim and Beyene [49]	2015	Amhara	community based	531	639	97.60%	9	83% (80,86)
Tessema et al. [50]	2019	pastoral/semi-pastoral	community based	255	600	96.60%	9	43% (39,47)
Kidane et al. [51]	2019	pastoral/semi-pastoral	community based	256	600	97%	9	43% (39,46)
Porth et al. [52]	2019	SNNP	community based	174	232	100%	6	75% (69,80)
EDHS [53]	2011	National	Survey	470	1930	97%	10	24% (22,26)
EDHS [16]	2016	National	Survey	781	2004	98%	10	39% (37,41)
EDHS [15]	2019	National	Survey	443	1026	99%	10	43% (40,46)

### Subgroup analysis

**Fig. 3:**
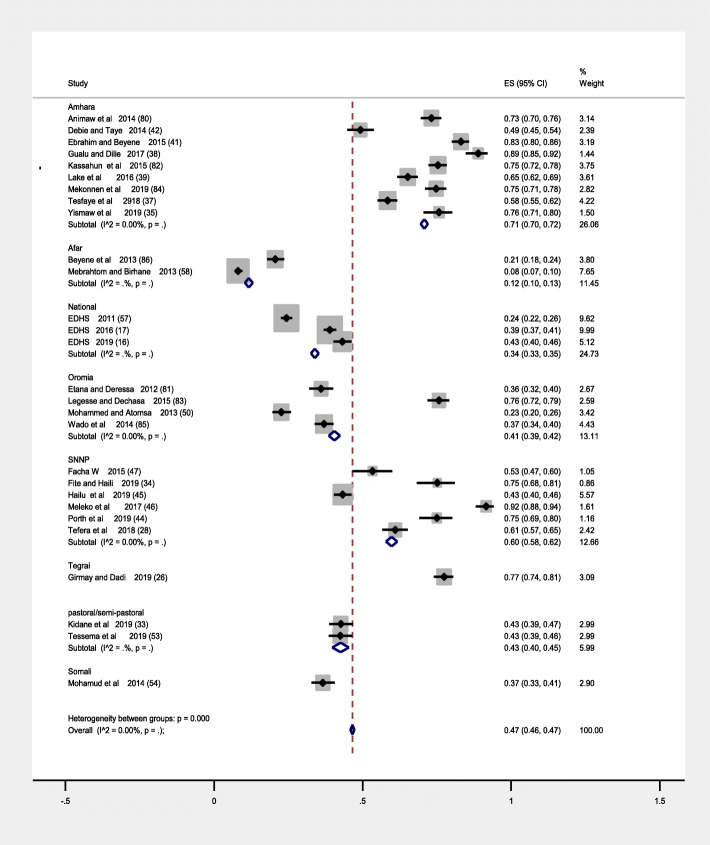
Forest plot subgroup prevalence of immunization coverage in Ethiopia from 2009 to 2019

We have conducted subgroup analysis based on regional states of the country, Tigrei region had highest prevalence of immunization coverage of 12–23 months children 77% (95% CI: 74.0,81.0) followed by Amhara region 71% (95% CI: 70.0,72.0), SNNP 60% (95% CI: 58.0,62.0), Oromia region 41% (95% CI: 41.0,42.0), Somali region, 37% (95% CI: 33.0,41.0) and A far region had the smallest immunization coverage in Ethiopia 12% (95% CI: 10.0,13.0). In subgroup analysis Amhara region had the highest weight 26.06 the possible reason may be high number of studies done and included in that area and lowest weight was Somali region 2.90 (Fig. 3) (below).

### Publication bias

**Fig. 4:**
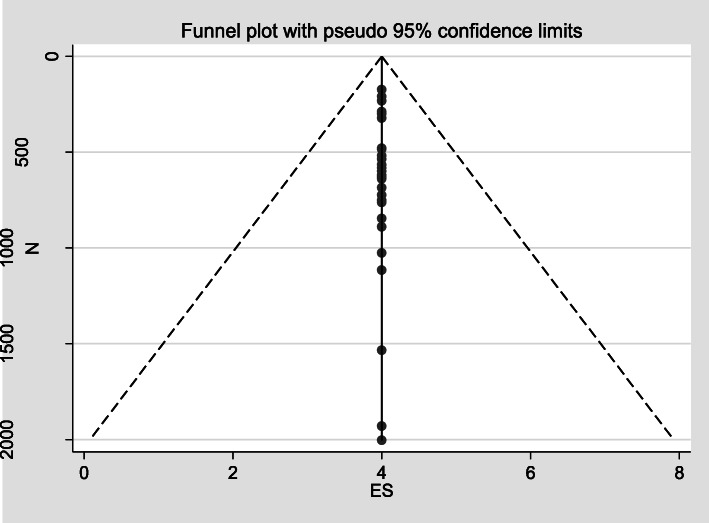
Forest plot subgroup prevalence of immunization coverage in Ethiopia from 2009 to 2019

In the present study there was no publication bias because studies were equally distributed within funnel plot, and visual inspection was resembled symmetrical (Fig. 4) (below).

## Discussion

To the best of our knowledge, the current meta-analysis is first of its kind for immunization coverage among 12–23 month old children in Ethiopia.

The government is still struggling to achieve its target of immunization coverage despite difficulties in implementing different strategies and approaches. In this systematic review and meta-analysis, we assessed pooled prevalence of immunization coverage among 12–23 month old children in Ethiopia.

Over all pooled prevalence of immunization coverage was 47.0% (95% CI: 46.0, 47.1). pooled prevalence is lower than the WHO 2018 factsheet report of 86% [10]. Similarly, it is lower than DHS reports of Lower and Middle Income Countries (LMIC), such as Kenya (67.2%) [56], Cameroon (62.5%) [57], Ghana (77.0%) [58], Tanzania (75.3%) [59], India (62%) [60], Philippines (70.0%) [61], Bangladesh (82%) [62], Zimbabwe (76.0%) [63] and South Africa (61.0%) [64]. In addition to that, this meta-analysis is also lower than HMIS (Health Management Information System) report of 88% and government target of health sector transformation plan-IV (HSTP) which is 93% [65].

Interestingly, the current work is in line with other DHS reports of Madagascar (49.1%) [66] and Uganda (55.8%) [67]. However, it is higher than DHS report and pooled prevalence of immunization coverage and associated factors which has been done in Nigeria were 31.0% [68] and 34.4% (CI: 27.0, 41.9) respectively [69]. We found regional variation in immunization coverage among 12–23-month children in Ethiopia. The lowest prevalence was observed from Afar region 12.0% (95% CI: 10.0, 13.0) while the highest was Amhara region that was 71% (95% CI: 70.1, 72.0).

There is variation among regional states of the country both Agrarian and pastoralists regions. The possible reason for high immunization coverage of some regions particularly agrarian communities may be they can easily have access to immunization service, have relatively better infrastructure, good socio-economic status, high literacy rate, access to information [70, 71]. The other reason may be women’s decision making, women who had medium wealth status and above, short distance to the health facility, good knowledge about complication during pregnancy, good perception toward immunization were positive contributing elements of immunization coverage [72–74].

On the other hand, regions that had low immunization coverage according to this finding were more likely to be pastoralists and semi-pastoral regions of the country accordance with evidences from EDHS 2011,2016 and mini 2019 [15, 16, 53]. Possible reason for low immunization coverage may be 82.12% of populations who are living in rural area face difficulties in having access to health services delivery this problem result in high mortality rate among under-fives particularly under-immunized and unimmunized children that is attributed to immunization [75].

Other problems include weak infrastructure, limited distribution and poor quality services such problem hindered access to health facility [76].

Still there are challenges that need special consideration particularly in pastoral and semi-pastoral regions of Ethiopia when it comes to routine immunization during planning programs. Special strategies and approaches are needed to ensure to have access and proper utilization of immunization services.

## Limitation

Despite carefully extensive search and planned reviews which were done, and more than two reviewers involved in minimizing all possible risk of bias, our study is not without limitation. Some of the limitations include the fact that we have reviewed only cross-sectional studies which are prone to confounding the number of studies which were not equally distributed among regional states. Only English language articles were synthesized, some articles were published in emerging journals, and numbers of studies included in the current study were very few and may affect the overall result.

## Conclusion

The current work found that pooled prevalence of immunization coverage among 12–23-month old children was very low compared to both international and national immunization coverage prevalence. Therefore, we recommend government to strength mobile and outreach immunization services for hard to reach and under developed pastoral and semi-pastoral regions. Secondly, we recommend integrating both human and animal health services provision due to the nature of the pastoral and semi pastoral communities. Thirdly; we recommend integrating immunization service in non-governmental health facilities since they account for 11% of health coverage of the country but hardly provide free immunization service. Lastly, future research should be considered for all states of the country that may reveal more about prevalence of immunization coverage and factors affecting immunization coverage should be synthesized.

## Data Availability

The dataset for this study is available from the corresponding author on reasonable request.

## Abbreviations

BCG: Bacillus Calmette Guerin
CI: Confidence Interval
DHS: Demographic Health Survey
DPT: Diphtheria Pertussis Tetanus
EDHS: Ethiopia Demographic Health Survey
EPI: Expanded Program of Immunization
HMIS: Health Management Information System
IRIS: Institutional Repository for Information Sharing
JBI: Joanna Briggs Institute
LMIC: Low and Middle Income Countries
PRISMA: Preferred Reporting Items of Systematic Reviews And Meta-Analysis
SDG: Sustainable Development Goals
VPDs: Vaccine Preventable Diseases
WHO: World Health organization
